# Chromatin-RNA in situ Reverse Transcription Sequencing (CRIST-seq) Approach to Profile the Non-coding RNA Interaction Network

**DOI:** 10.21769/BioProtoc.4718

**Published:** 2023-07-20

**Authors:** Shilin Zhang, Xue Wen, Lei Zhou, Hui Li, Wei Li, Andrew R. Hoffman, Ji-Fan Hu, Jiuwei Cui

**Affiliations:** 1 Key Laboratory of Organ Regeneration and Transplantation of Ministry of Education, Cancer Center, First Hospital of Jilin University, Changchun, Jilin 130061, China; 2 Stanford University Medical School, VA Palo Alto Health Care System, Palo Alto, CA 94304, USA

**Keywords:** CRIST-seq, Noncoding RNA, RNA-seq, Regulatory element, RNA–DNA interaction, Epigenetics

## Abstract

Non-coding RNAs (ncRNAs) are defined as RNAs that do not encode proteins, but many ncRNAs do have the ability to regulate gene expression. These ncRNAs play a critical role in the epigenetic regulation of various physiological and pathological processes through diverse biochemical mechanisms. However, the existing screening methods to identify regulatory ncRNAs only focus on whole-cell expression levels and do not capture every ncRNA that targets certain genes. We describe a new method, chromatin-RNA in situ reverse transcription sequencing (CRIST-seq), that can identify all the ncRNAs that are associated with the regulation of any given gene. In this article, we targeted the ncRNAs that are associated with pluripotent gene Sox2, allowing us to catalog the ncRNA regulation network of pluripotency maintenance. This methodology is universally applicable for the study of epigenetic regulation of any genes by making simple changes on the CRISPR-dCas9 gRNAs.

Key features

This method provides a new technique for screening ncRNAs and establishing chromatin interaction networks.

The target gene for this method can be any gene of interest and any site in the entire genome.

This method can be further extended to detect RNAs, DNAs, and proteins that interact with target genes.

Graphical overview

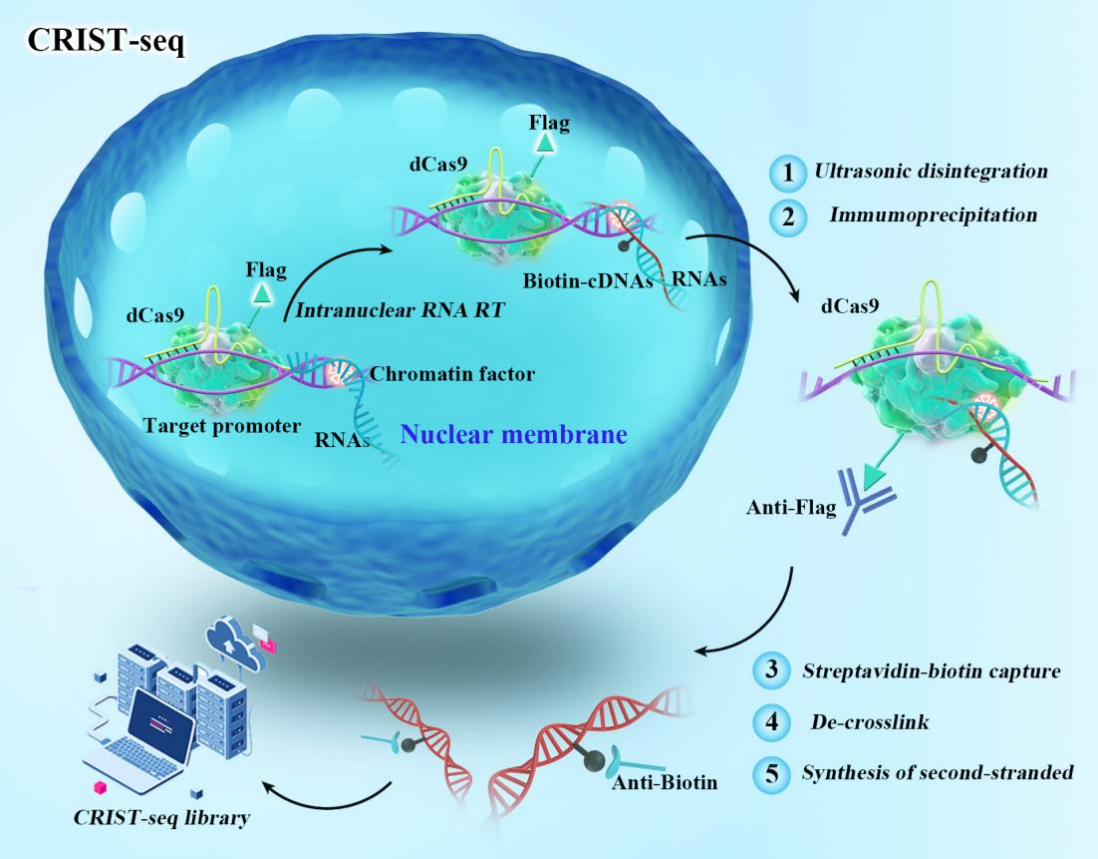

## Background

Non-coding RNAs (ncRNA), which include long non-coding RNA (lncRNA), small nuclear RNA, small nucleolar RNA, micro RNA, and other unknown RNA, play a critical role in the regulation of gene expression through multiple molecular mechanisms (Huang et al., 2016; Sridhar et al., 2017). RNA chips were initially used to identify RNAs; later, single-cell RNA sequencing expanded the ability to catalog the RNAs transcribed in a cell (Sajti et al., 2020). However, the existing RNA screening methods only focus on whole-cell RNA expression (Liang et al., 2019; Nicot, 2020) and are not sensitive enough to capture every known (or heretofore unknown) ncRNA that targets an individual gene.

To profile the ncRNA regulatory network at a specific gene locus, we developed a chromatin RNA in situ reverse transcription-associated sequencing (CRIST-seq) assay (S. Zhang et al., 2019). CRIST-seq combines the simplicity of intranuclear in situ reverse transcription–associated biotin labeling of ncRNA molecules with the specificity of catalytically inactive CRISPR dCas9 gene targeting. The assay includes 1) targeting a given gene using lentiviral dCas9-gRNAs, 2) nuclear in situ labeling of ncRNAs by reverse transcription with biotin-dCTP, 3) pulldown of chromatin-associated cDNAs by Cas9-Flag immunoprecipitation, 4) isolation of the target gene–associated cDNAs with streptavidin beads, and 5) Illumina cDNA library sequencing. We have used this approach to identify several novel ncRNAs that regulate the pluripotency of stem cells through various molecular mechanisms, including *Platr10* (Du et al., 2021), *Oplr16 (Oct4* promoter-interacting long noncoding RNA) (Jia et al., 2020), *Peblr20 (Pou5F1* enhancer binding lncRNA 20) (C. Wang et al., 2020), *Osblr8 (Oct4-Sox2* binding long noncoding RNA 8) (Zhu et al., 2020), *Osilr9 (Oct4-Sox2* interacting lncRNA 9) (Zhu et al., 2023), and *Peln1* (pluripotency exit lncRNA 1) (Y. Wang et al., 2022).

In this paper, we have used the *Sox2* gene, a critical stem cell factor required for pluripotent reprogramming, as an example to study and verify the feasibility of this method. This CRIST-seq approach may be broadly used to map ncRNA interaction networks at target loci anywhere in the genome by creating gene-specific gRNAs. CRIST-seq can be used to identify new ncRNAs and to study how these regulate gene expression in normal, stem, and cancer cells.

## Materials and reagents

**Consumables and reagents**

1. 6-well cell culture plates (Corning, catalog number: 3516)
2. 100 mm cell culture dishes (BD Falcon, catalog number: 353003)
3. 100 mm Petri dishes (BD Falcon, catalog number: 351005)
4. 1.5 mL microcentrifuge tubes (Pierce, catalog number: 69715)
5. 0.2 mL 8-tube stripes (Bio-Rad, catalog number: TBC0803)
6. 15 mL centrifuge tubes (Labselect, catalog number: CT-012-15)
7. DH5α competent *E. coli* (NEB, catalog number: ST10018)
8. Chloroform (Fisher Chemical, catalog number: C298-500)
9. Isopropanol (Thermo Scientific, catalog number: AC610080040)
10. Ethanol (Thermo Scientific, catalog number: 10617864)
11. Phenol/Chloroform/Isoamyl Alcohol (25:24:1) (Fisher Bioreagents, catalog number: BP1752I-100)
12. Glycogen (5 mg/mL) (Invitrogen, catalog number: AM9501)
13. RNase inhibitor (Invitrogen, catalog number: AM2694)
14. Random hexamers (Invitrogen, catalog number: N8080127)
15. dNTP mix (2.5 mM) (Invitrogen, catalog number: R72501)
16. Hybrid-Q Plasmid Rapidprep (GeneAll, catalog number: 100-102)
17. FastDigest Restriction endonucleases Age I (BshT I), Not I, and DpnI (Thermo Scientific, catalog numbers: FD1464, FD0596, and FD1703)
18. 10× FastDigest buffer (Thermo Scientific, catalog number: B64)
19. T4 DNA ligase (5 U/μL) (Thermo Scientific, catalog number: EL0011)
20. PEG-it virus precipitation solution (System Biosciences, catalog number: LV825A-1)
21. PEI (Polysciences, catalog number: 24765-1)
22. Polybrene (Sigma, catalog number: TR-1003)
23. Formaldehyde, 37% by weight (Fisher Chemical, catalog number: F79-500)
24. 2 M glycine (Sigma, catalog number: G8898)
25. NP-40 (Thermo Scientific, catalog number: 85124)
26. HEPES (Sigma, catalog number: H3375)
27. 0.5 M EDTA (Thermo Scientific, catalog number: R1021)
28. 10% SDS (Sigma, catalog number: L3771)
29. Halt protease inhibitor cocktail (100×) (Thermo Scientific, catalog number: 87785)
30. PMSF protease inhibitor (Thermo Scientific, catalog number: 36978)
31. Triton X-100 (Thermo Scientific, catalog number: A16046AE)
32. Tween 20 (Thermo Scientific, catalog number: J20605AP)
33. Proteinase K (Invitrogen, catalog number: 25530049)
34. Dynabeads M-280 streptavidin (Invitrogen, catalog number: 60210)
35. Formamide (Sigma, catalog number: F9037)
36. Biotin-14-dCTP (Invitrogen, catalog number: 19518018)
37. Anti-Flag antibody (Sigma, catalog number: F1804)
38. Anti-IgG antibody (Abcam, catalog number: ab171870)
39. PureProteome Protein A/G mix magnetic beads (Millipore, catalog number: LSKMAGAG10)
40. RNase A (20 mg/mL) (Invitrogen, catalog number: 12091021)
41. AMPure XP beads (Beckman Coulter, catalog number: A63881)
42. NEBNext ChIP-Seq Library Prep Master Mix Set for Illumina (NEB, catalog number: E6240)
43. NEBNext Multiplex Oligos for Illumina Index Primers Set 1 & Set 2 (NEB, catalog number: E7335 and E7500)
44. Maxima reverse transcriptase (200 U/μL) (Thermo Scientific, catalog number: EP0743)
45. NEBNext mRNA Second Strand Synthesis Module (NEB, catalog number: E6111)
46. 1 M TRIS-HCl pH 8.1 (Biyuntian, catalog number: ST781)
47. 1 M TRIS-HCl pH 7.5 (Biyuntian, catalog number: ST775)
48. 1 M TRIS-HCl pH 8.5 (Biyuntian, catalog number: ST785)
49. KCl (Thermo Scientific, catalog number: AC418200025)
50. NaCl (Fisher Scientific, catalog number: 15915)
51. MgCl_2_ (Thermo Scientific, catalog number: FERR0971)

**Plasmids**

1. pMD2.G (Addgene plasmid, catalog number: 12259)
2. psPAX2 (Addgene plasmid, catalog number: 12260)
3. P-GreenPuro (SBI plasmid, catalog number: SI505A-1)
4. Lenti dCas9-mSox2-gRNA #1-2-puro (constructed in our lab, see Procedure)

**Cell culture and cell culture reagents**

1. HEK293T cells (ATCC, catalog number: CRL-3219), stored in liquid nitrogen
2. Mouse FIB cells (muscle-derived fibroblasts), cultured from a 129 mouse fetus (Zhai et al., 2015) and stored in liquid nitrogen
3. Mouse induced pluripotent stem cells (iPSCs) were reprogrammed by Pou5f1-*Sox2*-Klf4-Myc (OSKM) cocktail factors in our lab using FIB cells (M. Chen et al., 2012; H. Zhang et al., 2013; Zhai et al., 2015; X. Chen et al., 2016), and stored in liquid nitrogen
4. KnockOut DMEM (Gibco, catalog number: 10829018)
5. KnockOut SR (Gibco, catalog number: 10828010)
6. DMEM (Gibco, catalog number: 11995-065)
7. FBS (Gibco, catalog number: A31605)
8. MEM-NEAA (Gibco, catalog number: 11140050)
9. Glutamine (Sigma, catalog number: G7513)
10. Penicillin-streptomycin (Sigma, catalog number: P4458)
11. β-mercaptoethanol (Sigma, catalog number: M3148)
12. ESGRO Mouse LIF (Millipore, catalog number: ESG1107)
13. Puromycin (Invivogen, catalog number: ant-pr-1)
14. Mitomycin C solution (1 mg/mL) (Nacalai Tesque, catalog number: 20898-21)
15. 2% gelatin solution (Sigma, catalog number: G1393)
16. PBS (Gibco, catalog number: 10010023)
17. Trypsin-EDTA (0.05%), phenol red (Gibco, catalog number: 25300054)
18. Opti-MEM (Gibco, catalog number: 51985034)
19. Knockout growth media (see Recipes)
20. Normal growth media (see Recipes)

**Primers**

1. See Table 1
  **Table 1.** List of primers or products

Primer/Product	5′→3′ sequence
pSox2-gRNA1	GGGGTTGAGGACACGTGCTG
pSox2-gRNA2	GAGCCAATATTCCGTAGCAT
gCT1	GTTCCCTGCAAGAGTGCCCA
gCT2	GCACTACCAGAGCTAACTCA
JH3915	TAGTAATGAGTTTAAACAAGGTCGGGCAGGAAGAGGGCCT
JH3986	CCAGCACGTGTCCTCAACCCCGGTGTTTCGTCCTTTCCACAAG
JH3987	GGGGTTGAGGACACGTGCTGGTTTTAGAGCTAGAAATAGCAAGTT
JH3988	ACATGCTACGGAATATTGGCTCGGATCCAAGGTGTCTCATACAG
JH3989	GAGCCAATATTCCGTAGCATGTTTTAGAGCTAGAAATAGCAAGTT
J441	CAACTTCTCGGGGACTGTGGGCGAT
pSox2-F	GAGCCAATATTCCGTAGCATG
pSox2-R	CGCTGGGGAACCTTTGTATC
5′CT-F	GAGCCAATATTCCGTAGCATG
5′CT-R	CGCTGGGGAACCTTTGTATC
Off-target-F	AGCCATCCTGTCCTCCGCCTG
Off-target-R	CTGCACGGAAGGTCACGATG

**Solutions**

1. Hypotonic buffer (see Recipes)
2. Sonication buffer (see Recipes)
3. ChIP dilution buffer (see Recipes)
4. Protein A/G beads binding & washing buffer (see Recipes)
5. Protein A/G beads elution buffer (see Recipes)
6. 2× M-280 beads binding & washing buffer (see Recipes)
7. M-280 beads elution buffer (see Recipes)

## Recipes

1. **Knockout growth media**
  KnockOut DMEM supplemented with 15% KnockOut SR
  1% MEM-NEAA
  2 mM glutamine
  1% penicillin-streptomycin
  200 μM β-mercaptoethanol
  ESGRO Mouse LIF
2. **Normal growth media**
  DMEM supplemented with 10% FBS and 1% penicillin-streptomycin
3. **Hypotonic buffer**
  10 mM HEPES
  1.5 mM MgCl_2_
  10 mM KCl
  0.4% NP-40 (add before use)
4. **Sonication buffer**
  50 mM Tris-HCl (pH 7.5)
  5 mM EDTA
  0.5% SDS
  1× Halt protease inhibitor cocktail (add before use)
  0.5 mM PMSF protease inhibitor (add before use)
5. **ChIP dilution buffer**
  0.01% SDS
  1.1% Triton X-100
  1.2 mM EDTA
  16.7 mM Tris-HCl (pH 8.1)
  167 mM NaCl
  1× Halt protease inhibitor cocktail (add before use)
6. **Protein A/G beads binding & washing buffer**
  0.05% Tween 20
  PBS, pH 7.4
7. **Protein A/G beads elution buffer**
  0.2 M Glycine-HCl, pH 2.5
8. **2× M-280 beads binding & washing buffer**
  10 mM Tris-HCl (pH 7.5)
  1 mM EDTA
  2 M NaCl
9. **M-280 beads elution buffer**
  10 mM EDTA (pH 8.2)
  95% formamide

## Equipment

1. Centrifuge (Eppendorf, model: 5415D)
2. Vibra-Cell Ultrasonic liquid processors (Sonics, model: VCX-130)
3. Thermomixer (Eppendorf, catalog number: vwrCA21516-176)
4. DNA spectrophotometer (NanoDrop, model: ND-1000)
5. Laboratory inverted microscope (Carl Zeiss, model: Axiovert 40 CFL)
6. pH meter (Mettler Toledo, model: SevenEasy S20)
7. PCR instrument (Eppendorf, model: Mastercycler pro PCR System)
8. Electrophoresis power supply (Pharmacia Biotech, model: EPS 300)
9. Labquake shaker (Lab Industries, catalog number: 400-110)
10. Gel documentation system (Axygen, model: GD-1000)

## Software

1. FASTX (Cold Spring Harbor Laboratory, http://hannonlab.cshl.edu/fastx_toolkit/)
2. TopHat (Center for Computational Biology at Johns Hopkins University, http://tophat.cbcb.umd.edu)
3. Cufflinks (Cole Trapnell’s lab at the University of Washington, http://cole-trapnell-lab.github.io/cufflinks/)
4. UCSC Genome Browser (UCSC Genomics Institute, https://genome.ucsc.edu)
5. RIPSeeker software (University of Toronto, http://www.bioconductor.org/packages/2.12/bioc/html/RIPSeeker.html)

## Procedure

- **Cell culture**
  1. HEK293T culture
    Grow HEK293T cells (2 × 10^6^) in a 6-well plate and culture in normal growth media (see Recipe 2) at 37 °C and 5% CO_2_. Use 293T cells to prepare lentivirus.
  2. FIB culture
    Grow FIB cells (1 × 10^7^) in a 100 mm plate and culture in normal growth media (see Recipe 2) at 37 °C and 5% CO_2_. Use FIB cells as control cells of iPSC.
  3. iPSC culture
*Note: Feeder cells can enhance the growth and reproduction of iPSCs, maintain pluripotency, and inhibit differentiation. In this assay, the feeding layer is made with FIB cells. After being treated with mitomycin, FIBs lose their mitotic ability and will die during digestion and inoculation, which has no impact on subsequent experiments.*
    - Coat a 100 mm plate with 2 mL of 0.1% gelatin and place in an incubator at 37 °C for 2 h.
    - Wash the plate twice with PBS and discard PBS before use.
    - Seed FIB cells (served as feeder layer) on the gelatin-coated plate and grow to ~70% confluency.
    - Add mitomycin C (final concentration of 10 μg/mL) and incubate for 2 h to block mitosis.
    - Wash the plate twice with PBS.
    - Grow iPSCs (1 × 10^6^) on feeder layer and culture in knockout growth media (see Recipe 1) at 37 °C and 5% CO_2_.
- **Lenti-dCas9 construction (Figure 1A)**
  1. Design gRNAs using Broad Institute website (https://portals.broadinstitute.org/gppx/crispick/public).
  2. Synthesize pU6-gRNA1-pH1-gRNA2 cassette using JH3915-JH3986, JH3987-JH3988, and JH3989-J441 primers (1 cycle at 95 °C for 5 min, 32 cycles at 95 °C for 20 s, 62 °C for 30 s, and 72 °C for 15 s, and 1 cycle at 72 °C for 10 min).
  3. Use Age I and Not I restriction enzymes (37 for 30 min) and T4 DNA ligase (22 for 10 min) to insert Sox2 gRNAs or gCTs downstream from U6 promotor in the lenti-dCas9 vector.
  4. Use DH5α competent cells for transformation.
  5. Confirm the correct cloning by sequencing and extract the plasmid with GeneAll Hybrid-Q Plasmid Rapidprep.
    **Figure 1.**
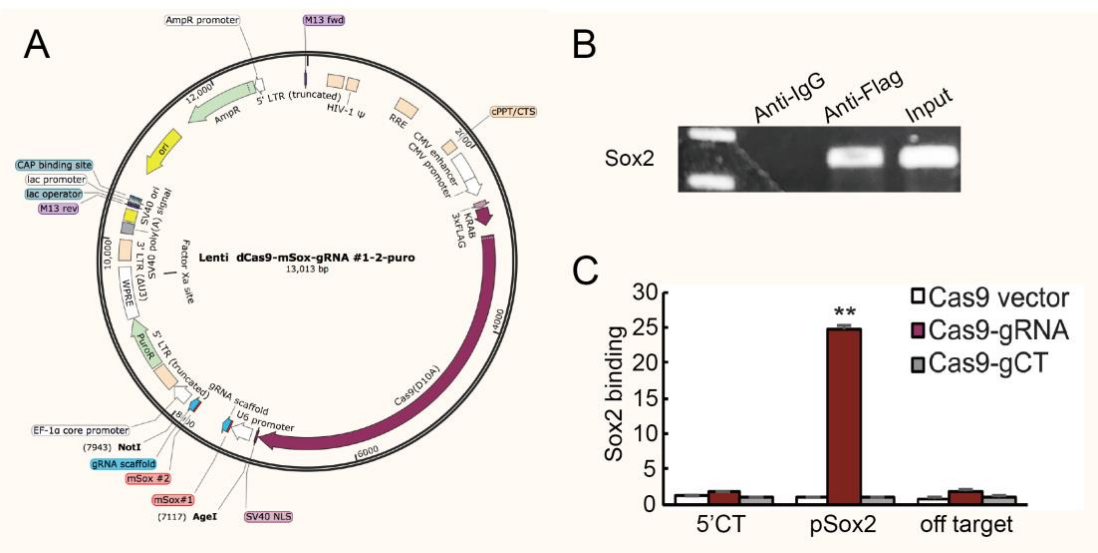
Plasmid maps and sample preliminary detection. (A) Map of lenti-dCas9 plasmid. Sox2-gRNA1 and Sox2-gRNA2 were inserted into the vector using Age I and Not I. (B) Preliminary detection of gRNA specific binding sites in chromatin-RNA in situ reverse transcription (CRIST) samples by PCR (using pSox2-F and pSox2-R primers). The sequence of gRNA specific binding sites cannot be detected in anti-IgG sample but can be detected in anti-Flag sample and input control, which demonstrates the specificity of the method. The anti-IgG here served as background control. (C) Sample specific testing was performed by real-time PCR using specific primers derived from the pSox2 targeting site, 5′-Ct control site, and off-target site. pSox2: targeting site in the Sox2 promoter where the Cas9 gRNAs are designed; 5′-Ct: fragment that is 14.6 kb away from the pSox2 target site and is used as the control site. Cas9 vector: cells that were treated with the Cas9 control vector that lacks the gRNAs; Cas9-gRNA: cells that were targeted by both Cas9 and Sox2 gRNAs; Cas9-gCT: cells that were treated with the random control gRNA vector. Off-target: CRIST control site that is 33.8 kb upstream of the housekeeping gene GAPDH. All data shown are mean ± SEM from three independent experiments by normalization over the IgG control. (∗∗) P < 0.01 as compared with the Cas9 Vector and Cas9-gCT controls.
- **Lentiviral packaging and transfection**
  1. Grow 293T cells to ~70% confluency for lentiviral packaging.
  2. Change the normal growth media to Opti-MEM 1h before packaging.
  3. Put 5 μg of PEI and 100 μL of Opti-MEM into a tube. Add 0.8 μg of psPAX2, 0.4 μg of PMD2.G, and 0.8 μg of lenti-dCas9 (carrying pSox2-gRNAs or gCTs).
  4. Mix and let stand for 20 min; then, add them to the culture media of 293T cells.
  5. Change to normal growth media (see Recipe 2) 6 h later.
  6. Collect virus supernatants at 24, 48, and 72 h, add PEG-it to the collected medium, incubate overnight at 4 °C, and spin at 1,500× *g* for 30 min.
  7. Discard the supernatant and resuspend viral particles in 100 μL of PBS.
  8. Grow iPSCs and fibroblasts (FIB) in 100 mm plates to ~50% confluency.
  9. Mix 20 μL of lentivirus solution with polybrene (final concentration of 5–10 ng/μL) and add it to culture medium.
  10. Change medium after 24 h and use puromycin (0.5–2 μg/mL) to select puromycin-resistant cells.
- **Intranuclear RNA reverse transcription**
*Note: In order to protect unstable RNAs, we performed intranuclear in situ reverse transcription prior to the subsequent steps and labeled RNAs with biotin-dCTP during reverse transcription.*
  1. Collect iPSCs and fibroblasts (~1 × 10^7^), resuspend with 1 mL of PBS, and add 54 μL of 37% formaldehyde (final concentration of 2%) for 10 min at room temperature. Quench the formaldehyde with 60 μL of 2 M glycine (with a final concentration of 0.125 M) and incubate for 5 min at room temperature.
  2. Wash both iPSCs and fibroblasts with PBS twice, resuspend with 800 μL of hypotonic buffer (see Recipe 3), and incubate for 5 min on ice to lyse the cell membranes.
  3. Centrifuge samples for 10 min at 1,500× *g* and wash twice with hypotonic buffer (without NP-40).
  4. Conduct intranuclear RNA reverse transcription at 65 °C for 30 min in a 20 μL reaction with biotin-dCTP (1 μL of 50 μM random hexamer, 1 μL of 10 mM dNTP, 1 μL of 0.4 mM biotin-dCTP, 1 μL of Maxima reverse transcriptase, 0.5 μL of RNase inhibitors, 1 μL of 0.1 M DTT, 4 μL of 5× cDNA synthesis buffer, and RNase-free water to 20 μL). Use 4 μL of 0.5 M EDTA to stop the reaction.
  5. Centrifuge samples at 1,600× *g* for 10 min at 4 °C. Wash twice with ice-cold PBS.
- **Ultrasonic disintegration**
  1. Resuspend cell pellets with 300 μL of sonication buffer (see Recipe 4).
*Note: Using too much liquid may lead to splashing, and using too little liquid may produce foam.*
  2. Clamp the sample tubes and the ultrasonic probe together and place them on ice.
  3. Put the 2 mm ultrasonic probe 1 cm below the liquid level.
*Note: In order to avoid foaming, the probe should always be kept below the liquid level*
  4. Shear DNAs into segments of 200–1,000 base pairs for 15 min (10 s on and 20 s off) at an amplitude of 40%.
  5. Divide 300 μL of sonicated DNAs into three portions (149, 149, and 2 μL). Use the two equal portions for immunoprecipitation (using anti-Flag and anti-IgG antibody respectively) and the 2 μL portion for input control.
- **Immunoprecipitation of biotin-cDNA and dCas9**
  1. To reduce non-specific background, use Protein A/G mix magnetic beads (5 μL/ChIP) to pre-clear the sonicated samples.
    - Put 5 μL of protein A/G beads in a new tube and wash twice with beads binding & washing buffer (see Recipe 6).
    - Dilute sonicated samples (10-fold) with ChIP dilution buffer (see Recipe 5), mix with 5 μL of washed protein A/G beads, and incubate at 4 °C with rotation for 30 min.
    - Discard protein A/G magnetic bead pellet in a magnetic rack and transfer sonicated samples to new tubes.
  2. Add anti-Flag and anti-IgG antibodies (2–5 μg/ChIP) to experimental and control groups, respectively, and incubate overnight at 4 °C with rotation.
  3. To enrich the immunoprecipitation samples, add 10 μL of pre-cleared protein A/G magnetic beads to samples and incubate at room temperature with rotation for 30 min. Wash the beads three times with Protein A/G Beads binding & washing buffer.
  4. Discard the supernatant and elute the immunoprecipitation samples twice with 100 μL of elution buffer (see Recipe 7).
- **Streptavidin-biotin capture**
  1. Put M-280 streptavidin beads in a new tube and wash twice with beads binding & washing buffer (see Recipe 8).
  2. Transfer 200 μL of eluent samples to the washed beads, add 50 μL of 5 M NaCl (final concentration of 1 M), and incubate for 30 min with rotation at room temperature.
  3. Discard the supernatant, elute the samples with 100 μL of elution buffer (see Recipe 9) twice, and collect 200 μL of eluate.
- **De-crosslink chromatin complex**
  1. Transfer 200 μL of eluate into a new tube.
  2. Neutralize the elution buffer with 20 μL of 1 M Tris-HCl (pH 8.5).
  3. Incubate samples and the input at 70 °C for 60 min with 20 μL of 5 M NaCl, 10 μL of 0.5 M EDTA, 20 μL of 1 M Tris-HCl (pH 7.5), 2 μL of 10 mg/mL Proteinase K, 2 μL of RNase inhibitor, and 226 μL of RNase-free water using an Eppendorf thermomixer.
*Note: This step can also be used to extract proteins for identification and analysis. This is an extended use of this method.*
  4. Purify cDNAs: Mix samples with equal volume of phenol/chloroform/isoamyl alcohol (25:24:1) and stand for 30 min at 4 °C. Centrifuge at 1,600× *g* for 10 min at 4 °C, collect supernatant, mix with equal volume of chloroform, add 2 μL glycogen, and stand for 30 min at 4 °C. Centrifuge at 1,600× *g* for 10 min at 4 °C, precipitate DNAs with 0.6× volumes of isopropanol for 30 min at 4 °C, and wash with 70% (v/v) ethanol.
- **Preliminary detection of CRIST samples**
*Note: In order to identify ncRNAs binding to the Sox2 gene promoter, we selected two sites from the Sox2 gene promoter to design gRNAs. Therefore, the ds-cDNAs obtained by the above method must contain the binding site of gRNAs on the Sox2 gene promoter, so that the ncRNA combined with it can be precipitated together, and the sample quality can be preliminarily determined as qualified and used for subsequent experiments.*
  Resuspend purified samples in 30 μL of ultrapure water and use pSox2 primers (Table 1) to detect the promotor binding site in anti-Flag and anti-IgG samples by PCR (1 cycle at 95 °C for 5 min, 28 cycles at 95 °C for 20 s, 60 °C for 15 s, and 72 °C for 15 s, and 1 cycle at 72 °C for 10 min), and use input DNA as positive control (Figure 1B). Cas9 enrichment signals were quantitated by real-time PCR using specific primers derived from the pSox2 targeting site, 5′-Ct control site, and off-target site (Figure 1C). After confirming the specificity of the Cas9 gRNA, the Cas9 Sox2-gRNA iPSCs were then used for CRIST-seq assay.
- **Synthesis of double-stranded cDNA**
  For long-term stability, the single-strand cDNAs should be converted into double-stranded cDNAs.
  1. Synthesize ds-cDNAs at 16 °C for 2.5 h with 20 μL of purified samples, 2 μL of synthesis enzyme mix, 4 μL of 10× reaction buffer, and 14 μL of RNase-free water. Use NEBNext mRNA Second Strand Synthesis Module.
  2. Purify DNAs again use phenol/chloroform/isoamyl alcohol (25:24:1) as previously described.
- **Construction of CRIST-seq library**
  1. To facilitate sequencing, use DpnI to cut cDNAs into fragments of approximately 300 bps: add 1 μL of Fastdigest DpnI and 3 μL of 10× FastDigest Buffer to 26 μL of purified cDNAs and incubate at 37 °C for 20 min.
  2. Purify DNAs with phenol/chloroform/isoamyl alcohol (25:24:1) and resuspend in 22 μL of ultrapure water.
    Follow the protocol of NEBNext ChIP-Seq Library Prep Master Mix Set for Illumina to prepare samples for next-generation sequencing.
*Note: Follow the protocol step by step from 1.1-1.8A.*
  3. Use Index primers in NEBNext Multiplex Oligos for Illumina Index Primers Set 1 & Set 2 to amplify samples and get the cDNA libraries (1 cycle at 98 °C for 30 min, 15 cycles at 98 °C for 10 s and 65 °C for 75 s, and 1 cycle at 65 °C for 5 min, then hold at 4 °C).
  4. Sequence the cDNA library and use index primers as sequencing tags.
- **CRIST-seq data analyses (Figure 2)**
  1. Filter raw data and the low-quality data using FASTX software (v0.0.13) (Du et al., 2018).
  2. Use TopHat software (version 2.0.9) to map clean reads to the mouse mm10 genome (Trapnell et al., 2009).
  3. Quantitate the mapped reads as “fragments per kilobase of transcript per million fragments mapped” (FPKM) using Cufflinks (version 2.1.1) (Trapnell et al., 2010).
  4. Call and annotate the peak using RIPSeeker software (Li et al., 2013) and adjust over the peaks overlapping with the IgG control enriched regions.
  5. Normalize the CRIST-seq signal intensities over the nontargeting Cas9 gCT control using the DiffBind package (Ross-Innes, Stark et al. 2012) [fold change difference ≥ 2 and p-value < 0.05, with false discovery rate (FDR) < 0.1].
    **Figure 2.**
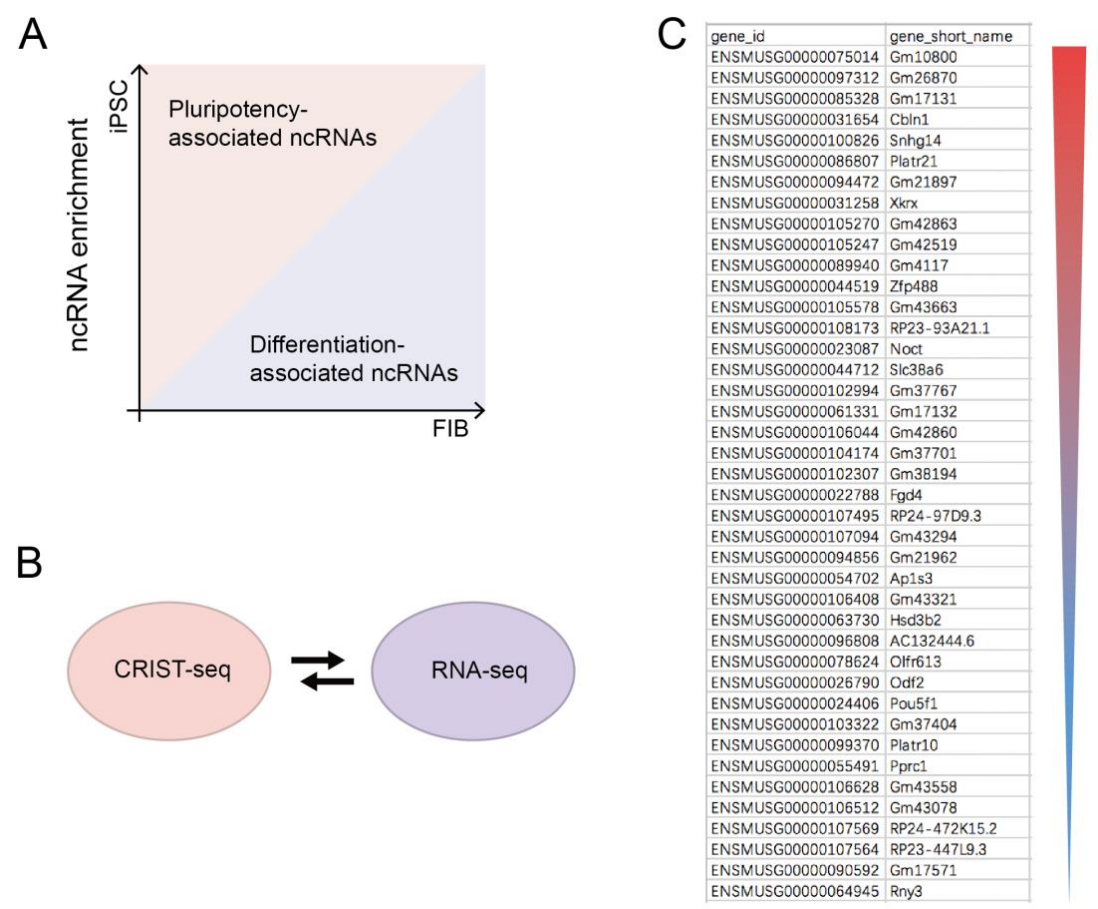
Sequencing analysis. (A) Analysis of ncRNA sequencing results. Preliminary screening for non-coding RNAs (ncRNAs) that are highly expressed in iPSC and much less expressed in FIB, which are related to pluripotency maintenance. Data regarding the RNAs that were changed greater than two-fold were combined with the CRIST-seq data using a VENN program. A cut-off threshold of peak enrichment FPKM > 50 was arbitrarily set to select CRIST-seq RNAs for VENN analysis. (B) Cross matching sequencing results with RNA-seq. (C) CRIST-seq identifies the top 42 Sox2 promoter-interacting RNAs. The Sox2 interacting RNAs are listed in order of the enrichment fold of the top 42 CRIST-seq data.
